# Deep learning application for abdominal organs segmentation on 0.35 T MR-Linac images

**DOI:** 10.3389/fonc.2023.1285924

**Published:** 2024-01-08

**Authors:** You Zhou, Alain Lalande, Cédric Chevalier, Jérémy Baude, Léone Aubignac, Julien Boudet, Igor Bessieres

**Affiliations:** ^1^Department of Medical Physics, Centre Georges-François Leclerc, Dijon, France; ^2^Institut de Chimie Moléculaire de l’Université de Bourgogne (ICMUB) Laboratory, Centre National de la Recherche Scientifique (CNRS) 6302, University of Burgundy, Dijon, France; ^3^Medical Imaging Department, University Hospital of Dijon, Dijon, France; ^4^Department of Radiotherapy, Centre Georges-François Leclerc, Dijon, France

**Keywords:** deep learning, MR-Linac, nnUNet, MR images, automatic segmentation

## Abstract

**Introduction:**

Linear accelerator (linac) incorporating a magnetic resonance (MR) imaging device providing enhanced soft tissue contrast is particularly suited for abdominal radiation therapy. In particular, accurate segmentation for abdominal tumors and organs at risk (OARs) required for the treatment planning is becoming possible. Currently, this segmentation is performed manually by radiation oncologists. This process is very time consuming and subject to inter and intra operator variabilities. In this work, deep learning based automatic segmentation solutions were investigated for abdominal OARs on 0.35 T MR-images.

**Methods:**

One hundred and twenty one sets of abdominal MR images and their corresponding ground truth segmentations were collected and used for this work. The OARs of interest included the liver, the kidneys, the spinal cord, the stomach and the duodenum. Several UNet based models have been trained in 2D (the Classical UNet, the ResAttention UNet, the EfficientNet UNet, and the nnUNet). The best model was then trained with a 3D strategy in order to investigate possible improvements. Geometrical metrics such as Dice Similarity Coefficient (DSC), Intersection over Union (IoU), Hausdorff Distance (HD) and analysis of the calculated volumes (thanks to Bland-Altman plot) were performed to evaluate the results.

**Results:**

The nnUNet trained in 3D mode achieved the best performance, with DSC scores for the liver, the kidneys, the spinal cord, the stomach, and the duodenum of 0.96 ± 0.01, 0.91 ± 0.02, 0.91 ± 0.01, 0.83 ± 0.10, and 0.69 ± 0.15, respectively. The matching IoU scores were 0.92 ± 0.01, 0.84 ± 0.04, 0.84 ± 0.02, 0.54 ± 0.16 and 0.72 ± 0.13. The corresponding HD scores were 13.0 ± 6.0 mm, 16.0 ± 6.6 mm, 3.3 ± 0.7 mm, 35.0 ± 33.0 mm, and 42.0 ± 24.0 mm. The analysis of the calculated volumes followed the same behavior.

**Discussion:**

Although the segmentation results for the duodenum were not optimal, these findings imply a potential clinical application of the 3D nnUNet model for the segmentation of abdominal OARs for images from 0.35 T MR-Linac.

## 1 Introduction

For several years, linear accelerators (Linacs) with integrated Magnetic Resonance Imaging (MRI) has made MR-guided radiotherapy (MRgRT) possible, offering an alternative image quality for the treatment planning and delivery compared to traditional X-ray-based imaging (1, 2). MRI provides superior contrast for soft tissues, making it more suitable for imaging the abdominal organs (3). Consequently, the clinical use of MR-Linacs has been particularly focused on stereotactic body radiation therapy (SBRT) of abdominal tumors (4–7). Indeed, MR imaging allows to get directly a precise delineation of target volumes and organs at risk (OARs) without complementary exams. The MR imaging is also acquired daily with the same sequence and parameters as the simulation image for treatment adaptation. Nevertheless, the position and the shape of several abdominal organs are not fixed since they are submitted to different movements related to breathing, cardiovascular and gastrointestinal activity (8). Since MR imaging uses non-ionizing radiation, it can be conducted multiple times during the treatment to monitor patient movements (gating process) or to adjust OAR and target variations between treatment sessions (adaptive radiotherapy process). This enhances both the safety and quality of the treatment (1). These processes are especially relevant in the context of abdominal SBRT since the healthy tissues are highly radiosensitive (9, 10).

In our institution, MR-guided abdominal SBRT (including gating and adaptive RT) is performed with the MRIdian (Viewray Inc., Oakwood Village, USA) 0.35 T MR-Linac since 2019 (11, 12). The success of these treatments and the reduced toxicity highly rely on the exact definition of the different OARs (13, 14). Radiation oncologists generally follow the established guidelines to define the volume of interest (15–17). The common practice is to manually draw the contours of different organs on the MR images. Nevertheless, an inter and intra observer variability is often pointed out, especially according to the level of expertise (18) and this is a very time consuming step in the radiotherapy (RT) workflow (19, 20).

The development of artificial intelligence (AI) has already begun to reshape our world, offering unprecedented advancements in the health care sector. Particularly, deep learning (DL) techniques represented by Convolutional Neural Networks (CNN) have been widely applied in the field of medical imaging segmentation. Originating from a cell segmentation challenge, UNet network (21), with its main structure based on the encoder-decoder structure, is currently the most popular automatic segmentation method in the field of multi-organ segmentation (22). Many researchers have made improvements based on this foundational network that have been applied to abdominal segmentation. For example, Oktay et al. (23) applied attention mechanisms (originally used for natural language processing) to the UNet, which improved the accuracy of pancreas segmentation for CT (Computed Tomography) images. Sabir et al. (24) improved the segmentation of liver tumors for CT images using the ResUNet, by combining the attention mechanism, residual blocks and the UNet, together. Besides, the EfficientNet uses fixed coefficients to scale the network’s depth, width and resolution. It improves performance while reducing computational expense (25). Khalil et al. (26) replaced the backbone of the UNet with the EfficientNet and subsequently improved the performance segmentation of the OARs for abdominal CT images.

Despite the success of these UNet-based neural networks, the search for neural network hyperparameters and preprocessing or post-processing techniques still require a high level of knowledge and experience (27, 28). To face this problem, a fully automated segmentation framework designed for medical imaging called the nnUNet has been developed (28). The network structure and the training strategy can be automatically adjusted based on different data. Since its introduction, the nnUNet has achieved state-of-the-art results on many medical segmentation datasets from different medical imaging techniques. For instance, it achieved the first place in the 2019 Kidney and Kidney Tumor Segmentation (KiTS19) competition and fourth place in the Combined (CT-MR) Healthy Abdominal Organ Segmentation (CHAOS) challenge (29–31).

Within the realm of radiotherapy, AI has shown its capacity to aid radiation oncologists in tumor diagnosis and treatment (32, 33). For instance, Kawula et al. employed the 3D UNet for segmenting clinical target volume and OARs in the pelvic area, using MR images obtained from 0.35 TMR-Linacs, underscoring the potential of AI applications in MRgRT (34). In this context, we decided to investigate the automatizing of abdominal OARs segmentation on 0.35 T MR-Linac images in order to optimize the treatment workflow and its quality. The performances of the Classical UNet, the ResAttention UNet, the EfficientNet with the EfficientNet-b4 as its encoder and the nnUNet were investigated for the prediction of abdominal OARs from 0.35 T MR-Linac images. This work specifically focused on five OARs: the liver, the kidneys, the spinal cord, the stomach and the duodenum. The objective of this work was to find the most accurate automatic organ contouring model using the proposed DL techniques based on dedicated metrics.

## 2 Materials and methods

### 2.1 Data acquisition and preprocessing

A total of 121 series of abdominal axial MR images have been collected from 77 patients treated for liver cancer and 44 patients treated for pancreas cancer. The images have been acquired with our 0.35 T MRIdian MR-Linac (Viewray Inc., Oakwood Village, USA) device using a balanced steady-state free precession (SSFP, T2/T1-weighted) sequence during breath-hold. Five OARs have been considered for this study: liver, kidneys, spinal cord, stomach and duodenum. The delineations used for each treatment were also collected and reviewed by one expert radiation oncologist to be considered as the ground truth in this work. The updates included missing data or incorrect segmentation. Specifically, in the treatment of liver cancer, the radiation oncologists might only segment the kidney on the side closest to the liver tumor. The kidney on the other side was also segmented. Similarly, when the stomach is far from the liver tumor, they might only segment the half of the stomach closest to the tumor. The entire stomach was segmented. Additionally, the segmentation of the spinal cord by the radiation oncologists is often too coarse, typically several times its normal size. Although these segmentation ambiguities do not affect clinical treatment, they can impact the training process of the neural network. In consequence, these segmentations have been refined.

**Figure 1:**
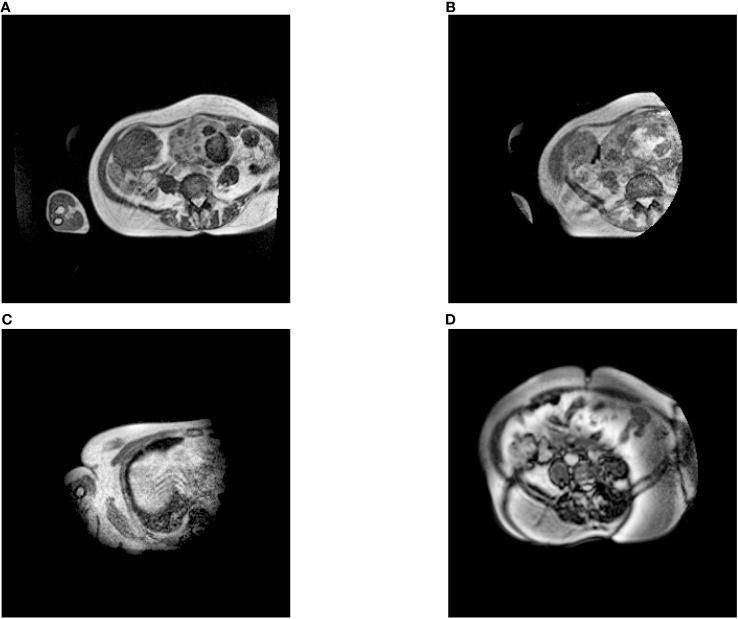
Examples of axial MR images from different exams. **(A)** is the image we kept, and **(B–D)** were removed. Specifically, **(B, C)** are only half exposed, while half of **(D)** is not clear.

**Figure 2:**
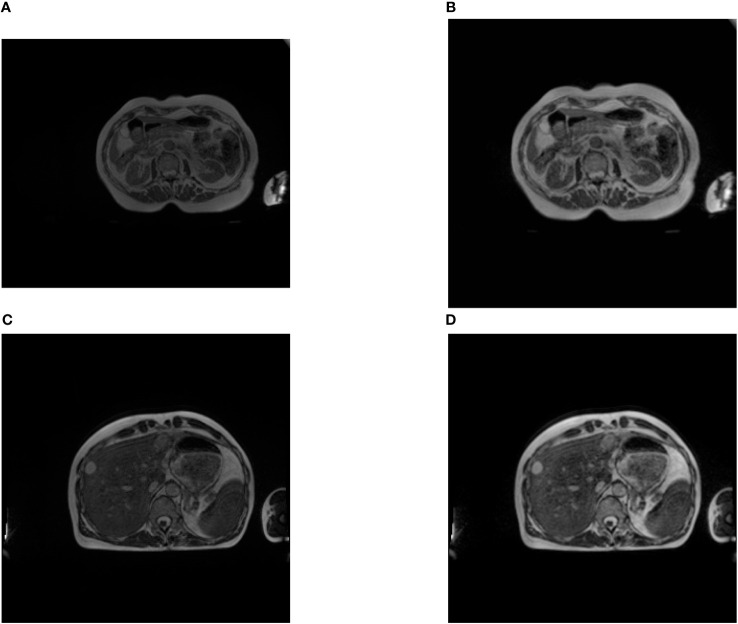
Examples of images before and after preprocessing **(A, C)** are the original images, **(B, D)** are the transformed images. We can see that the contrast of the images is enhanced. It can be observed that, after preprocessing, the originally rectangular image **(A)** has been cropped and transformed into a square one **(B)**.

The characteristics of the MR images from the 121 patients are displayed in **Table 1**. Due to the poor homogeneity of the magnetic field at the extremities of the field of view, as shown in **Figure 1**, higher levels of artefacts and distortion tend to be seen in these areas. Consequently, the corresponding 2D slices were discarded and the remaining 2D slices of the same patient were kept. Specifically, for images containing 80 2D slices, the first 3 slices and the last 3 slices were removed. For images with 140 2D slices, the first 19 slices and the last 47 slices were discarded. To ensure that the data input into the neural network has a consistent shape, the images were resampled from their original dimensions to a standardized size of 288 × 288 pixels. For images of size 310 × 360, their size was first cropped to a size of 310 × 310, and employed then bilinear interpolation was employed to resample them to a resolution of 288 × 288. For images measuring 310 × 310, bilinear interpolation was used to resample them to a size of 288 × 288. The nearest neighbor interpolation method was employed for resampling the corresponding masks. The second preprocessing technique involved was a limiting filtering to remove near-zero values from the background. Due to significant variations in brightness within certain images, the CLAHE (Contrast Limited Adaptive Histogram Equalization) method was employed to augment the contrast. Additionally, this method assists in diminishing noise intensity, obviating the need for alternative standardization techniques (35). Two pairs of images showing the difference before and after the preprocessing are displayed in **Figure 2**.

**Table 1:** Characteristics of images for the 121 patients.

Number of patients	Image height (number of pixels)	Image width (number of pixels)	Pixel size (mm²)	Slice thickness (mm)	Number of Slices
5	310	360	1.6 × 1.6	3.0	140
116	276	276	1.5 × 1.5	3.0	80

### 2.2 Data augmentation

**Figure 3:**
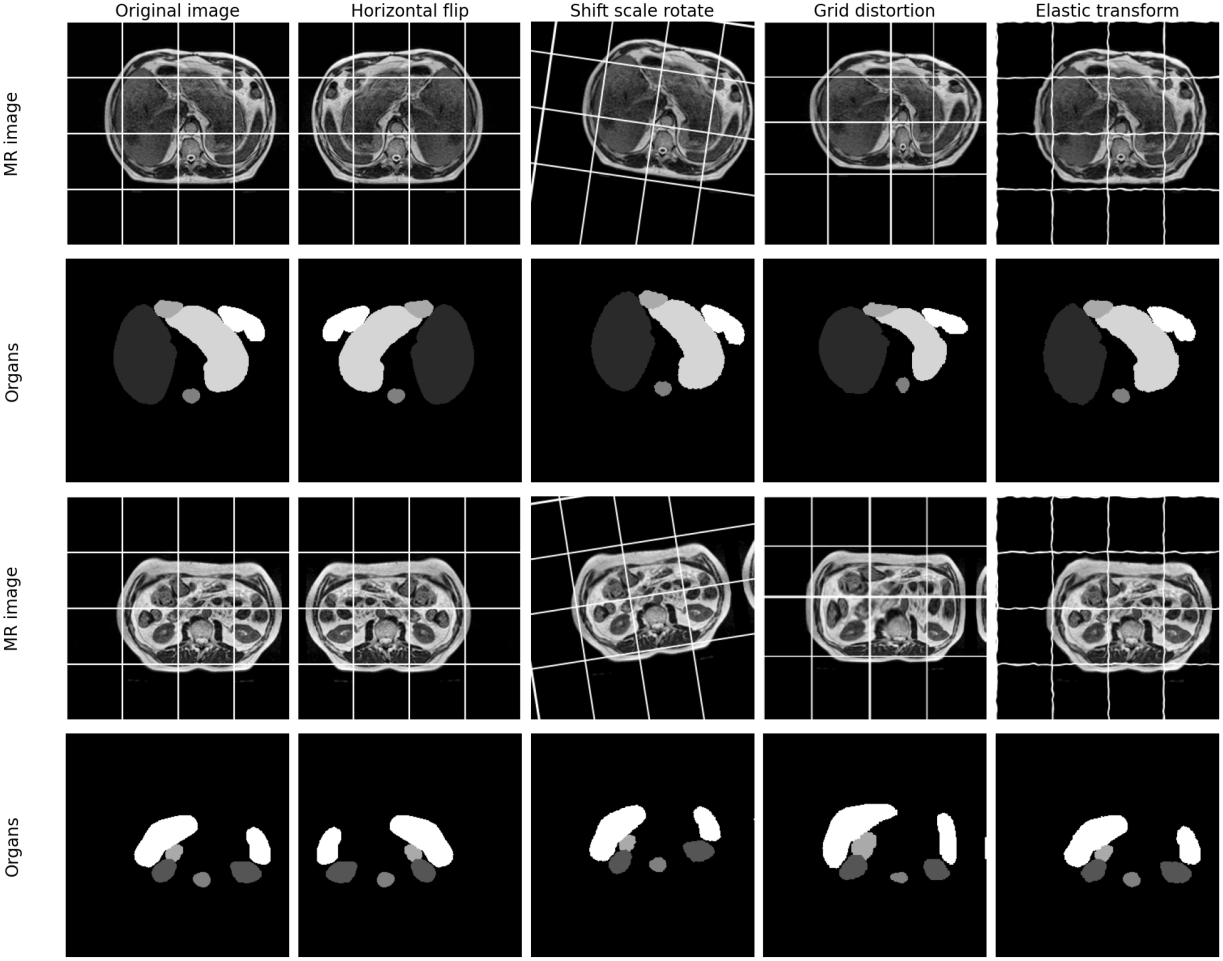
Examples of original images and the associated data augmentations. Gridlines are added to the image to better illustrate the results of the data augmentation. It can be observed that after grid distortion, the spacing between lines in the image has become non-uniform. After elastic transformation, the straight lines in the image appear curved.

Neural networks are prone to overfit when training images are insufficient (27). Data augmentation can increase the training samples by making minor modifications to the existing data. The techniques detailed in **Table 2** and illustrated in **Figure 3** were used in order to further augment our dataset. Among them, ‘grid distortion’ applies a grid over the image and then introduces random shifts to the grids’ edges. In contrast, ‘elastic transform’ starts by generating a random displacement field and then uses it to deform the image (36). Some techniques such as horizontal flipping provides images that are aberrant anatomically. However, on a relatively scarce dataset, it is preferable to do data augmentation with data that are not anatomically possible rather than not doing data augmentation. This process adds some noise in the data, and then, reinforces the network even if it appears not to be logically. The Albumentations library was used to augment our data. This library has been reported as a fast and flexible implementation (36).

**Table 2:** This table lists the augmentation techniques used in our method, their application probabilities to images before neural network input, and the associated parameters for each.

Augmentation	Probability of use	Parameters	Description
Horizontal Flip	0.5	–	Flip the picture horizontally
Shift scale rotate	0.5	shift limit = 0.0625 scale limit = 0.05 rotate limit = 10	Randomly apply affine transformations
Grid distortion	0.5	grid number = 5 distort limit = 0.05	Grid deformation of images
Elastic transform	0.5	alpha affine = 50 alpha = 1 sigma = 50	Elastic deformation of images

### 2.3 Automatic segmentation models: UNet and variations

**Figure 4:**
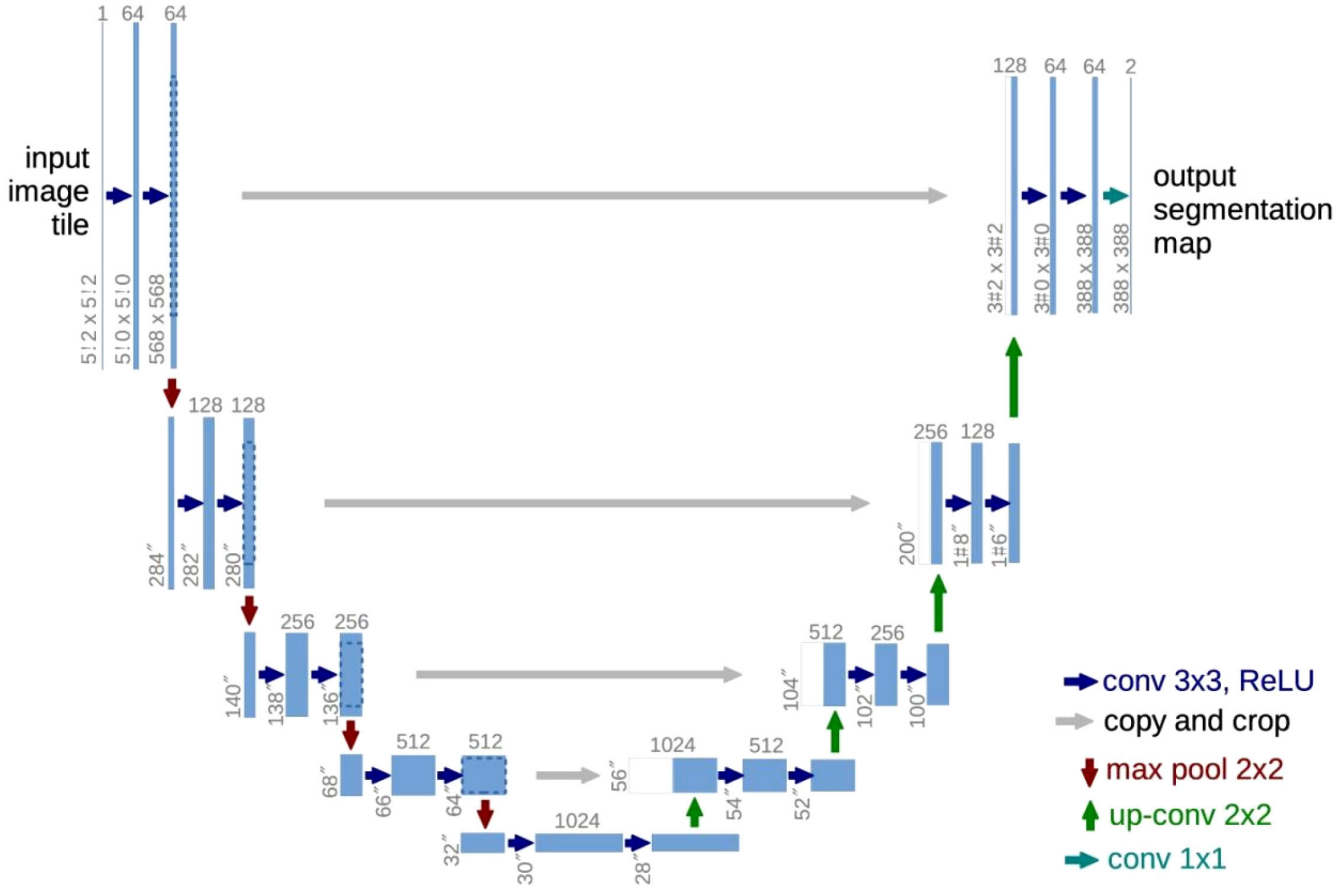
The structure of UNet. Modified from (21).

Four types of the UNet have been used in this study: the Classical UNet, the ResAttention UNet, the EfficientNet UNet and the nnUNet (21, 23–26). As depicted in **Figure 4**, the UNet employs an encoder-decoder structure with skip connections. Blue rectangles denote feature maps, and white rectangles indicate direct duplicates of the feature maps on the left. The encoder on the left is responsible for feature extraction, and the decoder on the right decodes the encoded information. With information acquired from the skip connections, the UNet can directly utilize spatial data for prediction. By integrating the ResNet as its backbone and adding an attention mechanism, a model called the ResAttention UNet can be derived. Similarly, when the encoder of the UNet is replaced with the EfficientNet, another variation of the UNet named the EfficientNet UNet can be defined.

For the classical UNet, the ResAttention UNet and the EfficientNet UNet, the following parameters have been used: training batch size to 16, the AdamW optimizer, an initial learning rate of 0.001. The learning rate was reduced to a minimum of 0.000001 using the reduce learning on plateau strategy, which divides the original learning rate by 5 when there is no improvement after eight consecutive epochs. The 5-fold cross-validation was used in the training set. The first three models require extensive experimentation by experienced researchers to identify the optimal hyperparameters. In this context, the nnUNet diverges from this approach not by altering the UNet architecture, but by automating the search for its training parameters. Initially, it processes the dataset to generate dataset fingerprints, which include characteristics such as image size and modality. Subsequently, it auto-configures parameters like batch size and patch size based on a set of rules. These parameters are then automatically integrated with pre-established blueprint parameters, including learning rate and loss functions, to generate pipeline fingerprints. The resulting pipeline fingerprints serve as the training specifications for the UNet model. The nnUNet, after analyzing our data, determined all the required parameters for training in both 2D and 3D modes, and then used these parameters to train the neural network. The nnUNet has integrated parameter search tasks, so it is not necessary to define loss functions, optimizers, and other hyperparameters like when training our first three models. Then for the training of the nnUNet, the source code provided by the authors was utilized. For each model, the same method of random splitting was employed to divide the dataset into a training set and a test set, containing 110 patients and 11 patients, respectively. Python 3.10 and PyTorch 2.0 were used to train the models.

### 2.4 Post-processing method

**Figure 5:**
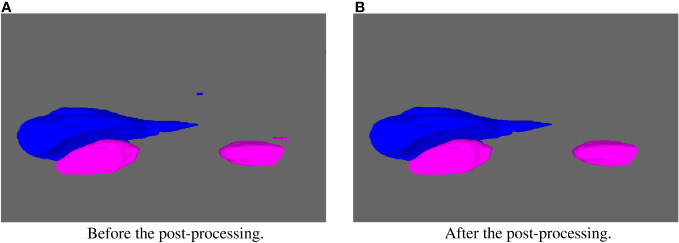
Example of the segmentation results for the liver and kidneys. **(A)** displays the result before the post-processing. **(B)** displays the result after the post-processing. The liver is in blue and the kidneys in pink.

In the example in the **Figure 5**, the segmentation results for the liver and kidneys contain some minor noise that is not connected to the main segmented structure. To solve this problem, post-processing technique based on 3D connected regions is commonly used in medical image segmentation and has yielded satisfactory results (29, 37). This method was applied to all the considered organs in our study. Specifically, for organs such as the liver, the spinal cord, the duodenum and the stomach, the largest connected region was retained. For the kidneys, both the largest and the second largest connected regions were kept.

### 2.5 Evaluation method

#### 2.5.1 Geometrical comparison

To evaluate the model performances, the Dice Similarity Coefficient (DSC) (**Equations 1**), Intersection over Union (IoU) coefficient (**Equations 2**), and Hausdorff distance (HD) (**Equations 3**) were calculated in 3D mode. The DSC and IoU coefficients allow us to determine the similarity of two sets based on the extent of their overlap. Their respective formulae are as follows:


(1) $$\begin{array}{ccc} \text{DSC} & = & \frac{2\;*\;\text{Overlap Volume}}{\text{Total Volume}}\\ & = & \frac{2\;*\;\text{Overlap Volume}}{\text{Predicted Volume}+\text{Ground truth Volume}} \end{array}$$

(2) $$\begin{array}{ccc} \text{IoU} & = & \frac{\text{Overlap Volume}}{\text{Union Volume}}\\ & = & \frac{\text{Overlap Volume}}{\text{Predicted Volume}+\text{Ground truth Volume}-\text{Overlap Volume}} \end{array}$$

The HD is employed to evaluate the distance between two volumes. Its formula is as follows:


(3) $$\text{HD}=\text{max}\left(\underset{x\in A^{y\in B}}{\sup\;\inf}d(x,y),\underset{y\in B^{x\in A}}{\sup\;\inf}d(x,y)\right)$$

In this equation: *A* and *B* represent the two sets of 3D points being compared. *d*(*x,y*) is the distance between points *x* and *y*. $\underset{x\in A^{y\in B}}{\sup\;\inf}d(x,y)$ calculates the largest of the smallest distances from each point in *A* to *B*. For each point *x* in *A*, it finds the nearest point in *B* (which is the minimum distance, represented by $\underset{y\in B}{\inf}d(x,y))$ . Then it finds the largest of these minimum distances (represented by $\underset{x\in A}{\sup}$ ). Similarly, $\underset{y\in B^{x\in A}}{\sup\;\inf}d(x,y)$ calculates the largest of the smallest distances from each point in *B* to *A*. The HD allows us to highlight local outliers. In order to eliminate the impact of a very small subset of the outliers, the 95th percentile of the Hausdorff distances (95HD) has also been considered.

Despite variations in pixel sizes across patients, our methodology ensures a consistent and robust calculation of the HD. Initially, the HD was computed using pixel units. Then, the specific pixel sizes of each MRI were considered. As illustrated in **Table 3**, which showcases HD values for all organs across all patients in the test set, the HD results for the patient 3 (with different pixel size) is aligned closely with those of other patients, indicating minimal impact of pixel size variations on our analysis.

**Table 3:** HD of the organs of all patients in the test set, where the pixel size of the third patient was different from that of the others.

Patient #	HD (mm)				
	Liver	Kidney	Spinal Cord	Duodenum	Stomach
1	12.5	12.3	3.8	98.5	99.6
2	9.8	16.5	3.8	21.9	11.9
3	11.3	7.2	3.1	45.8	23.4
4	13.4	13.7	5.1	11.4	33.0
5	30.7	30.9	5.1	12.3	9.2
6	7.6	14.5	3.8	22.1	26.9
7	11.5	19.3	5.1	53.0	100.8
8	12.1	21.0	4.2	30.1	8.7
9	12.1	13.7	4.2	69.2	28.0
10	12.4	7.0	4.8	56.2	22.2
11	13.8	14.5	2.4	51.8	17.8

#### 2.5.2 Volume comparison

The correlation coefficient (r) and the Bland-Altman plot were used to analyze the automated predicted organ volume and compare it to the one obtained with the manual ground truth. Contrary to geometrical metrics, considering an anatomical parameter such as the volume allows us to reach an usable metric in clinical practice. The correlation coefficient (r) shows how closely the volumes obtained with the manual ground truth and the predicted results are related, and consequently characterizes the stability of the model. On the other hand, the Bland-Altman diagram focuses on the agreement between these two measurements by calculating the average and standard deviation of the differences of both values and points out possible bias. This study of agreement can be displayed by a specific graph called the Bland-Altman plot (38, 39).

## 3 Results

The geometrical performances of the different models are displayed for each organ in **Table 4**. For each investigated model (the Classical UNet, the ResAttention UNet, the EfficientNet UNet, and the nnUNet trained in both 2D and 3D modes), the DSC, IoU and HD mean values of the test set have been calculated in 3D with the corresponding standard deviation.

**Table 4:** DSC, IoU, HD and 95HD values of the different tested models on the five OAR.

Model Name	Metrics	Liver	Kidney	Spinal Cord	Duodenum	Stomach
Classical UNet	DSC	0.92 ± 0.02	0.77 ± 0.06	0.87 ± 0.02	0.39 ± 0.13	0.59 ± 0.12
	IoU	0.86 ± 0.04	0.64 ± 0.08	0.77 ± 0.03	0.25 ± 0.10	0.43 ± 0.11
	HD (mm)	23.0 ± 7.5	26.0 ± 11.0	6.2 ± 3.5	45.0 ± 15.0	45.0 ± 22.0
	95HD (mm)	8.9 ± 3.7	16 ± 8.3	5.9 ± 4.9	32.0 ± 13.0	30.0 ± 22.0
ResAttention UNet	DSC	0.94 ± 0.02	0.82 ± 0.05	0.87 ± 0.02	0.39 ± 0.13	0.67 ± 0.09
	IoU	0.88 ± 0.03	0.70 ± 0.07	0.77 ± 0.04	0.25 ± 0.10	0.52 ± 0.10
	HD (mm)	21.0 ± 6.1	26.0 ± 15.0	6.0 ± 3.7	45.0 ± 14.0	42.0 ± 24.0
	95HD (mm)	7.0 ± 2.4	15.0 ± 14.0	4.1 ± 2.9	34.0 ± 12.0	27.0 ± 23.0
EfficientNet UNet	DSC	0.95 ± 0.01	0.88 ± 0.04	0.86 ± 0.03	0.43 ± 0.11	0.75 ± 0.09
	IoU	0.90 ± 0.02	0.78 ± 0.07	0.76 ± 0.04	0.28 ± 0.09	0.61 ± 0.10
	HD (mm)	17.0 ± 6.8	18.0 ± 8.0	5.5 ± 3.0	44.0 ± 15.0	43.0 ± 27.0
	95HD (mm)	5.4 ± 2.5	8.2 ± 5.0	6.0 ± 4.9	32.0 ± 13.0	27.0 ± 25.0
nnUNet 2D	DSC	0.95 ± 0.02	**0.91 ± 0.02**	0.90 ± 0.03	0.53 ± 0.25	0.82 ± 0.09
	IoU	0.91 ± 0.04	0.83 ± 0.04	0.83 ± 0.04	0.40 ± 0.24	0.70 ± 0.12
	HD (mm)	16.0 ± 11.0	16.0 ± 7.7	4.3 ± 2.1	46.0 ± 27.0	42.0 ± 31.0
	95HD (mm)	6.6 ± 6.6	6.5 ± 4.1	4.3 ± 4.5	36.0 ± 26.0	26.0 ± 30.0
nnUNet 3D	DSC	0.96 ± 0.01	0.91 ± 0.02	0.91 ± 0.01	0.69 ± 0.15	0.83 ± 0.10
	IoU	**0.92 ± 0.01**	**0.84 ± 0.04**	**0.84 ± 0.02**	**0.54 ± 0.16**	**0.72 ± 0.13**
	HD (mm)	**13.0 ± 6.0**	**16.0 ± 6.6**	**3.3 ± 0.7**	**42.0 ± 24.0**	**35.0 ± 33.0**
	95HD (mm)	**3.8 ± 0.7**	**6.3 ± 3.8**	**2.2 ± 0.3**	**32.0 ± 23.0**	**23.0 ± 30.0**

Each value is presented with its standard deviation. The values in bold correspond to the best performances.

**Figure 6:**
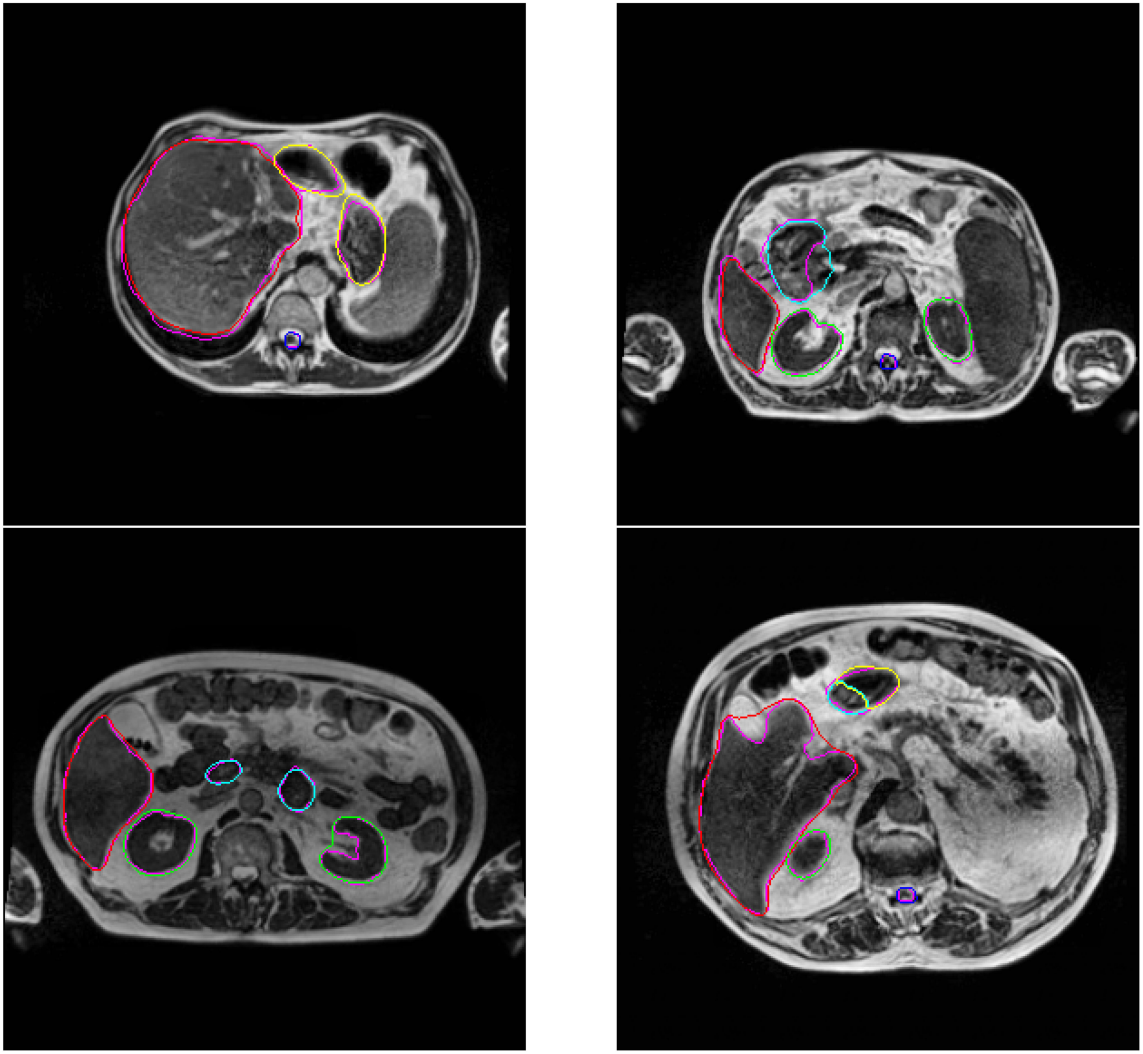
2D image examples of the display of the segmentation done by the 3D nnUNet. The segmented organs liver, kidneys, spinal cord, stomach and duodenum are in red, green, blue, cyan and yellow. The ground truth of each organ is in purple.

**Figure 7:**
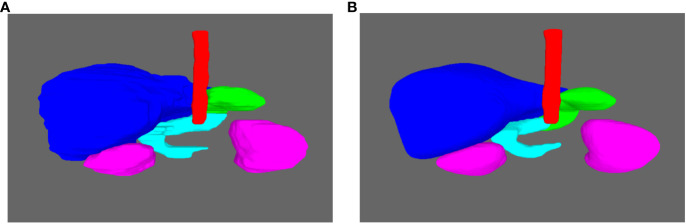
Examples of the 3D display of the segmention done by the 3D nnUNet. **(A)** The ground truth. **(B)** The obtained segmention with the nnUNet 3D. The liver, kidneys, spinal cord, stomach and duodenum are in blue, pink, red, cyan and green.

An improvement of the results is observed through these geometrical metrics for each organ by complexifying the UNet network. The nnUNet trained in 2D mode outperformed other 2D networks across all organs. As all the models have been trained with 2D strategy, considering the results in 2D, only the nnUNet was also been trained with 3D strategy. There is further improvement when the nnUNet was trained in 3D mode. The behavior of the 3D nnUNet results is illustrated in **Figures 6**, **7**. For the liver, the kidneys and the spinal cord, the mean DSC is particularly high (*>* 0.91) with a very limited standard deviation (*<* 0.02). For the stomach, the mean DSC is lower, but remains at a relatively satisfying level (0.83). For the duodenum, the mean DSC is even lower and the values are below 0.69. Similar tendencies have been observed for the IoU and the HD. Moreover, the tested neural network underperfomed for the duodenum and the stomach considering the HD. This may be attributed to the difficulty of the neural network in discerning the boundaries of the duodenum and the stomach.

Further volumetric analysis has been done for 3D nnUNet based on the correlation coefficient and the Bland-Altman plot (available in **Supplementary Material**) and the results are summarized in **Table 5**. For liver, kidneys and spinal cord, a high level of correlation and a good agreement between the considered volumes confirms the stability and the accuracy of the model. For the duodenum and the stomach, the correlation coefficient is very low demonstrating nonsystematic behavior of the model. This is also illustrated with the high level of the standard deviation of the mean difference for both organs. However, according to the results of the Bland-Altman pots, the agreements remain acceptable compared with the mean absolute volume of the organ.

**Table 5:** Quantitative comparison of organ volumes: ground truth and nnUNet 3D segmentation results.

Organ	Correlation Coefficient (r)	Volume Difference (cm^3^) mean ± standard deviation	Mean from the Ground Truth (cm^3^)
Liver	0.99	0.07 ± 33.02	1164.65
Kidneys	0.95	3.30 ± 24.76	201.76
Spinal Cord	0.97	-0.02 ± 0.90	11.28
Duodenum	0.46	-14.00 ± 37.39	70.99
Stomach	0.36	4.43 ± 31.66	123.22

Metrics include correlation coefficient, mean volumetric difference, and standard deviation, as derived from Bland-Altman plots.

## 4 Discussions

**Figure 8:**
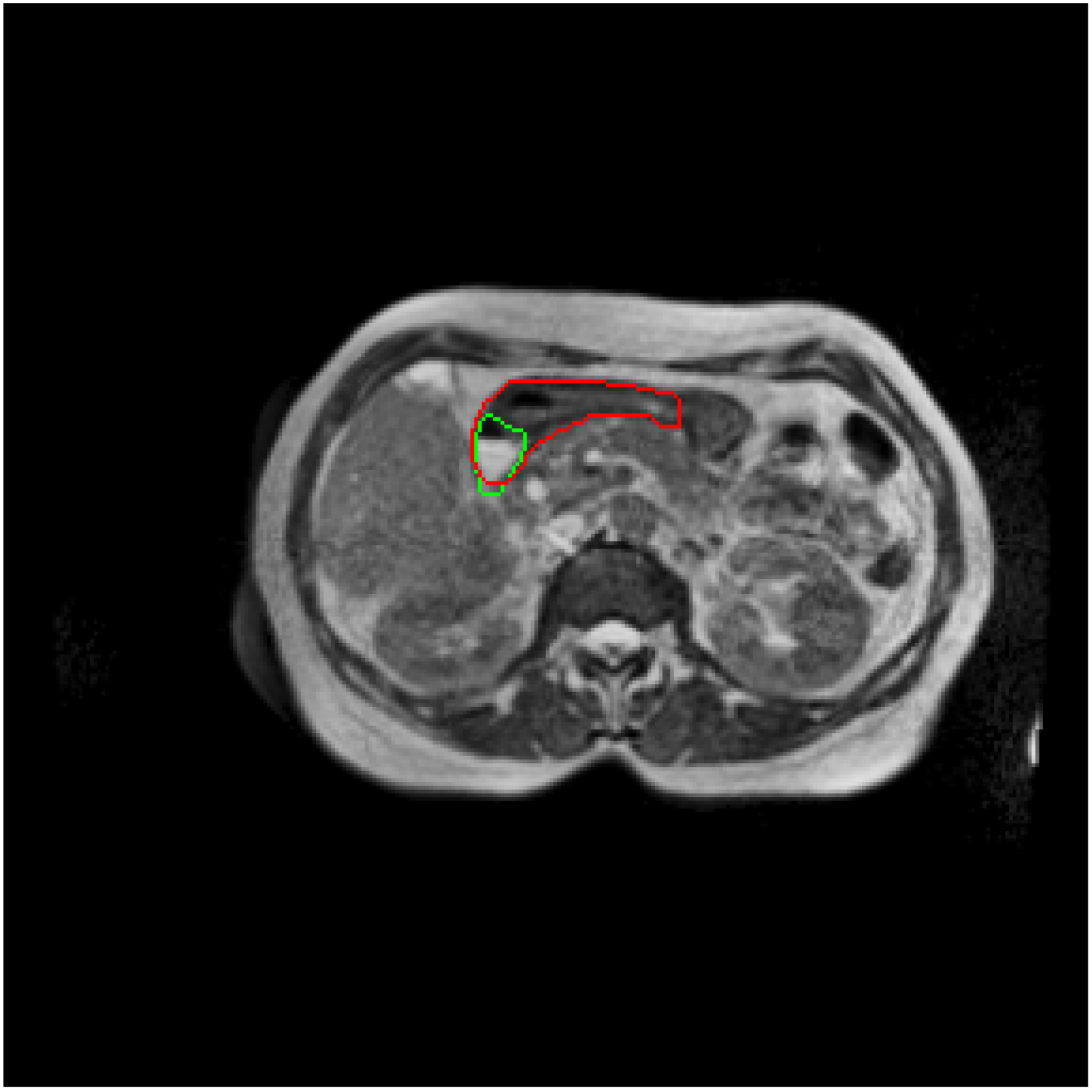
Manual segmentation of the duodenum. Segmentation of the same MR images by two different radiation oncologists.

In 2D, we found that the nnUNet outperformed the Classical UNet, the ResAttention UNet and the EfficientNet UNet for the segmentation of all the OARs. Notably, according to our knowledge, the nnUNet was used for the first time to do organ segmentation in the abdomen using a 0.35 T MR-Linac. Additionally, the 3D version of the nnUNet is more effective than the 2D version. It was not necessary to compare the 3D versions of all the networks as the ranking of the methods in 3D conforms to the one in 2D in most of the medical imaging segmentation tasks (22). We have observed that these models share the same limits in the segmentation of OARs and the results varied across different organs. Specifically, their performance in segmenting the duodenum and stomach was slightly inferior compared to their accuracy in delineating the liver, kidneys and spinal cord. Indeed, it is challenging to distinguish the junction between the stomach and the duodenum in MR images. As a consequence, a significant variability in the ground truth could impact the training and affect the prediction. We tried to highlight this issue by asking two radiation oncologists to contour independently the stomach and the duodenum of 11 patients. The DSC results between both radiation oncologists are displayed in **Table 6** and an example is shown in **Figure 8**. These results highlight the important variation in the segmentation task, especially for the duodenum. Nevertheless, one can observe that the DSC between both radiation oncologists for the cumulative volume of stomach and duodenum is at a very satisfying level and greater or equal to 0.8, reinforcing the assumption that the limit between both organs is difficult to determine and highly depends on the level of experience. Consequently, it is difficult to ensure that the ground truth used for the deep learning training represents the real organs and thus, that the models are able to detect them properly. By consolidating the duodenum and stomach predictions in the nnUNet 3D as a single structure, as illustrated in **Table 7**, an enhancement in prediction accuracy was observed compared to when these organs were considered independently. This suggests that the challenge in segmenting the duodenum and stomach lies in distinguishing their boundaries. The nnUNet DSC results for the duodenum and the stomach were better than those obtained from the radiation oncologists. The superior DSC results from the nnUNet can be attributed to the model’s consistency, which outperforms the inter-observer variability caused by different human observers.

**Table 6:** DSC results for segmentation of the duodenum, the stomach, and combine the two organs as one organ by two different radiation oncologists.

Patient #	Duodenum	Stomach	Duodenum + Stomach
1	0.39	0.66	0.87
2	0.55	0.83	0.88
3	0.33	0.70	0.84
4	0.35	0.81	0.88
5	0.48	0.86	0.86
6	0.71	0.87	0.88
7	0.66	0.92	0.86
8	0.68	0.87	0.86
9	0.45	0.76	0.86
10	0.43	0.78	0.88
11	0.44	0.73	0.80
Mean DSC	0.50	0.80	0.86

**Table 7:** DSC results for segmentation of the duodenum, the stomach, and combine the two organs as one organ by the ground truth and the result of nnUNet 3D.

Patient #	Duodenum	Stomach	Duodenum + Stomach
1	0.49	0.68	0.91
2	0.81	0.87	0.91
3	0.64	0.79	0.89
4	0.87	0.90	0.90
5	0.83	0.91	0.91
6	0.64	0.84	0.91
7	0.39	0.65	0.85
8	0.81	0.93	0.89
9	0.69	0.85	0.89
10	0.83	0.91	0.90
11	0.72	0.81	0.88
Mean DSC	0.70	0.83	0.89

Most automatic abdominal segmentation models in the literature focus on CT imaging. However, there are also studies on MR images, acquired either for diagnostic purposes or with an MR-Linac device. Fu et al. (40) used a CNN-based correction 3D network to segment abdominal organs on a 0.35 T MR-Linac. Compared with their approach, our segmentation of the duodenum was better (DSC: 0.69 vs 0.65), while the results for other organs were similar. Chen et al. (41) utilized a 2D UNet, replacing the UNet’s encoder with a Densely-connected Block, and analyzed images obtained from a 3.0 T MR device by inputting images from three different views: transversal, coronal, and sagittal. Their segmentation results for the duodenum and the stomach surpassed ours. Amjad et al. (42) used multi-sequence MR images acquired from 3.0 T MR device for training to segment abdominal organs, achieving better segmentation results for the kidneys, the duodenum and the stomach. These improvements might be attributed to their utilization of a diagnostic MR device avoiding MR-Linac possible artefacts (43), offering a higher magnetic field strength with a better image contrast and a training based on several MR contrasts.

It can take more than 20 minutes for a radiation oncologist to delineate the five OARs manually without the help of a deep learning model. In contrast, the nnUNet model we trained is able to automatically predict the five OAR in 16 seconds on a NVIDIA V100 32GB GPU. The predicted segmentation results for the five organs of the nnUNet allows us to consider it for clinical use, including a step for expert review post-prediction. While the predictions from the model still sometimes require refinement by radiation oncologists, the integration of this technology substantially reduces their workload and enhances the efficiency of radiation therapy (22). This time saving could be especially relevant during online adaptive radiotherapy for abdominal tumor on MR-Linacs, where the duration of the procedure is a crucial factor (44). The online implementation of DL-based automatic segmentation could help to improve this kind of treatment.

In addition, several limitations and perspectives have been identified in our study. First, the default training process of nnUNet was used without any fine-tuning, although further optimization could potentially enhance the results. Second, the ground truth definition of several organs could be improved by crosschecking the segmentation of different experts. Finally, owing to data limitations, only five organs for the prediction could be selected, but many other critical organs, such as the colon, bowel and esophagus could be included. Considering that less than 10% of the dataset for testing is controversial and an increase of the dataset would resolve this limitation.

## 5 Conclusion

In this study, we investigated the automatic segmentation of abdominal OARs on 0.35 T MR-Linac images using several UNet based model variations. The 3D nnUNet gave the best results achieving encouraging performance for a clinical use. The use of this kind of model could be of high interest especially for online adaptive radiotherapy to save time and limit operator variability. Several limitations have been pointed out in order to improve the prediction, especially the ground truth segmentation definition and validation.

## Data availability statement

The raw data supporting the conclusions of this article will be made available by the authors, without undue reservation.

## Ethics statement

Ethical approval was not required for the study involving humans in accordance with the local legislation and institutional requirements. Written informed consent to participate in this study was not required from the participants or the participants’ legal guardians/next of kin in accordance with the national legislation and the institutional requirements.

## Author contributions

YZ: Data curation, Formal analysis, Investigation, Software, Writing – original draft. AL: Conceptualization, Methodology, Project administration, Supervision, Validation, Writing – review & editing. CC: Data curation, Writing – review & editing. JBa: Validation, Writing – review & editing. LA: Writing – review & editing. JBo: Writing – review & editing. IB: Conceptualization, Methodology, Project administration, Supervision, Validation, Writing – original draft, Writing – review & editing.
