# Supplementary material for: Deep learning application for abdominal organs segmentation on 0.35 T MR-Linac images
Source: Front Oncol. 2024 Jan 8;13:1285924. doi: 10.3389/fonc.2023.1285924 (PMC10800957; doi:10.3389/fonc.2023.1285924)
Supplement: Supplementary file 1 [file DataSheet_1.pdf]

Supplementary Material

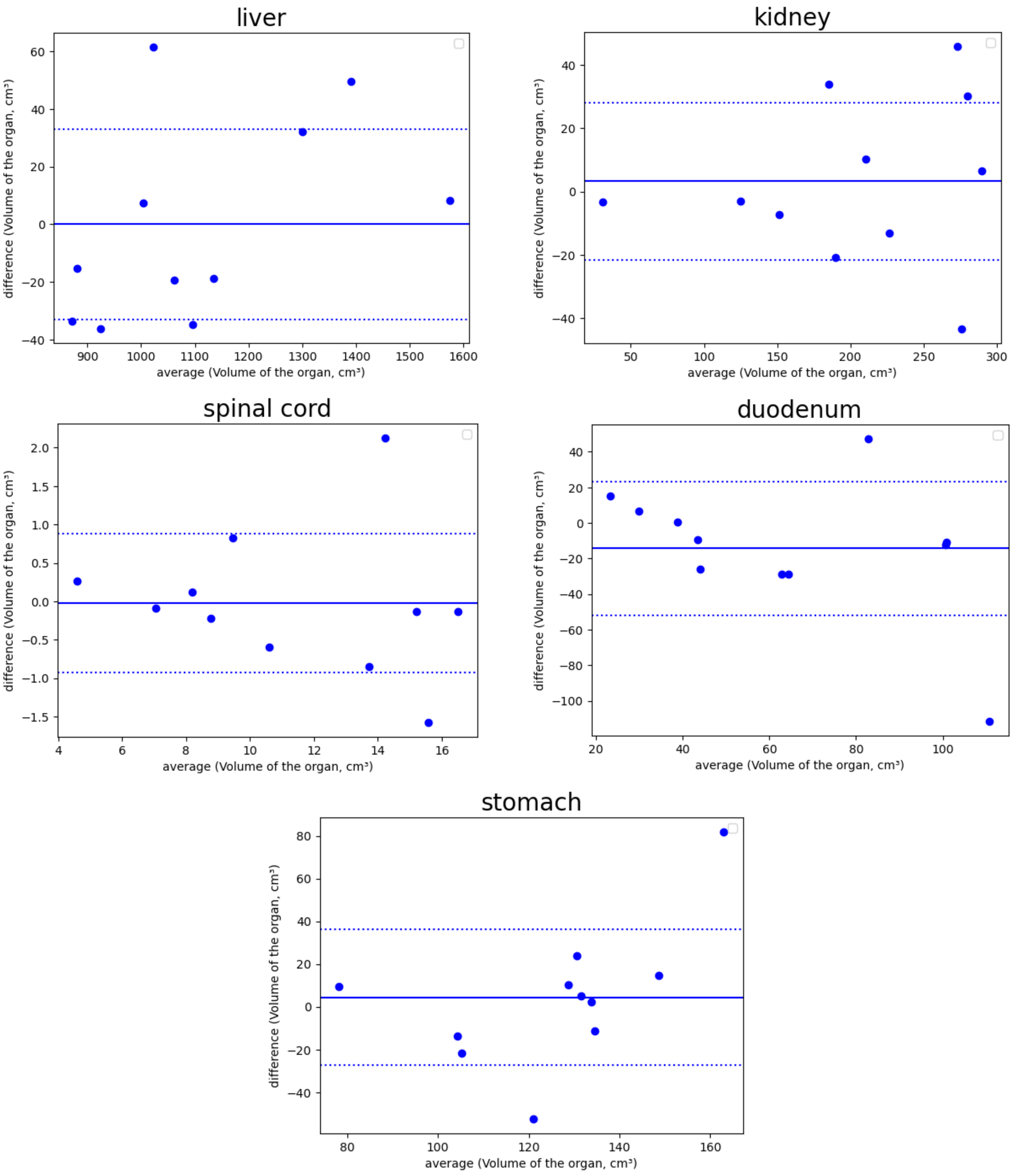

Figure S1. Bland-Altman plots for various organs predicted with nnUNet 3D.
